# Validation Framework for Sleep Stage Scoring in Wearable Sleep Trackers and Monitors with Polysomnography Ground Truth

**DOI:** 10.3390/clockssleep3020017

**Published:** 2021-05-03

**Authors:** Quyen N. T. Nguyen, Toan Le, Quyen B. T. Huynh, Arveity Setty, Toi V. Vo, Trung Q. Le

**Affiliations:** 1 Department of Medical Instrumentation, School of Biomedical Engineering, International University of Vietnam National University, Ho Chi Minh City, Vietnam; ngntquyen@gmail.com (Q.N.T.N.); huyn-hquyen.dtvt@gmail.com (Q.B.T.H.); vvtoi@hcmiu.edu.vn (T.V.V.); 2 Department of Biomedical Engineering, North Dakota State University, Fargo, ND 58108, USA; toan.le@ndsu.edu; 3 Department of Industrial and Manufacturing Engineerring, North Dakota State University, Fargo, ND 58108, USA; 4 Sanford Health, Fargo, ND 58102, USA; arveity.setty@sanfordhealth.org

**Keywords:** wearable device, polysomnography, sleep monitoring, sleep stage scoring, sleep stage assessment

## Abstract

The rapid growth of point-of-care polysomnographic alternatives has necessitated standardized evaluation and validation frameworks. The current average across participant validation methods may overestimate the agreement between wearable sleep tracker devices and polysomnography (PSG) systems because of the high base rate of sleep during the night and the interindividual difference across the sampling population. This study proposes an evaluation framework to assess the aggregating differences of the sleep architecture features and the chronologically epoch-by-epoch mismatch of the wearable sleep tracker devices and the PSG ground truth. An AASM-based sleep stage categorizing method was proposed to standardize the sleep stages scored by different types of wearable trackers. Sleep features and sleep stage architecture were extracted from the PSG and the wearable device’s hypnograms. Therefrom, a localized quantifier index was developed to characterize the local mismatch of sleep scoring. We evaluated different commonly used wearable sleep tracking devices with the data collected from 22 different subjects over 30 nights of 8-h sleeping. The proposed localization quantifiers can characterize the chronologically localized mismatches over the sleeping time. The outperformance of the proposed method over existing evaluation methods was reported. The proposed evaluation method can be utilized for the improvement of the sensor design and scoring algorithm.

## 1. Introduction

The emerging trend in transforming point-of-care sleep tracking devices to polysomnographic alternatives has necessitated methods to evaluate and validate sleep monitoring functions. Wearable devices with actigraphy sensors have been used widely for sleep monitoring, although their accuracy is still controversial [1,2]. It has been shown that even though actigraphy is a more approachable and accepted alternative to PSG for non-laboratory settings [3,4,5,6,7,8], sleep staging (i.e., annotating of N1, N2, N3, or REM stage) functions of actigraphy are still questionable [9]. In addition to actigraphy, wearable sleep tracking devices have recently been developed with additional sensors to record different physiological parameters during the sleeping time [10]. Concurrently, their performances have improved considerably [11]. However, when considering the detailed distribution of sleep stages consisting of all wake, light sleep, deep sleep, and REM stages, as well as the transitions among these stages during sleep, the current wearable sleep trackers do not show comparable results with those from the PSG gold standard [12]. As the point-of-care technologies in sleep monitoring offer more details on sleep staging beyond just sleeping and waking [13], such detailed information can be used for the diagnosis of many other sleep-related disorders [14,15,16,17]. An assessment method to compare the detailed distribution of sleep stages and quantify the transition among sleep stages between wearable sleep tracking devices and PSG is critical.

Although different validation tests have been proposed, very few (if any) works have investigated and characterized the localized mismatch of sleep staging in wearable sleep tracking devices. Previous studies to validate sleep tracking devices can be divided into three groups. The first group of methods utilized Tryon’s approach to compare the sensitivity, specificity, and overall accuracy of the correct sleep staging [18] generated by the wearable devices and PSG system. The sensitivity is the proportion of PSG-registered epochs, also identified as sleep stages by the sleep tracker devices, whereas specificity for sleep is the proportion of wake epochs correctly identified by the sleep tracker device. The second group of methods evaluates the differences in the overall sleep stages of multiple sleep tracking devices using statistical tests such as paired sample *t*-tests [19,20], the two-way repeated measure analyses of variance [18,20], and Bland–Altman plotting’s test [21]. The third group of methods evaluates the overall epoch-by-epoch correlations using the confusion matrix of Pearson correlations [18,19,22], Cohen’s kappa, and Fleiss’ kappa [23]. Those comparative methods provide only overall sleep staging comparisons without quantifying the transition mismatch among sleep stages and the monotone correlations among sleep episodes. Such an estimation fails to take into account the chance agreement because of the high base rate of sleep during the night and the interindividual difference across the sampling population. Furthermore, the previous comparisons were performed using different sleep stage classifications, which were usually defined subjectively by the sleep staging algorithms of the sleep tracker devices. Such an invariance of the sleep staging––relative to the American Academy of Sleep Medicine (AASM) sleep classification guideline [24]––among different sleep tracking devices hinders the accuracy of the interdevice validation study. Very few previous validation methods investigated temporal-ordered epoch-to-epoch disagreements between wearable sleep tracker devices and the PSG ground truth with the adjustment of AASM-based sleep classification.

This paper proposes a systematic framework to quantitively assess the performance of wearable sleep tracking devices relative to the PSG gold standard. The proposed framework not only characterizes the difference in the sleep stage distribution, but also quantifies the chronologically localized mismatches over the sleeping time. Three major components of the framework consist of AASM-based sleep stage classification, sleep staging distribution analysis, and localized comparison analysis. The proposed research extends our earlier work on evaluating commercial sleep trackers compared to PSG [11,25,26]. The proposed method was illustrated and validated on a set of different commonly used wearable sleep tracking devices. The rest of the paper is organized as follows: the Methodology Section describes the methodology of the general sleep feature evaluation, sleep stage evaluation, and localized quantifier estimation of the proposed evaluation framework. The validation of the proposed framework to evaluate four different wearable devices with sleep tracking functions is described in the Results Section. A discussion of the results and conclusions is presented in the last section.

## 2. Methodology

**Figure 1:**
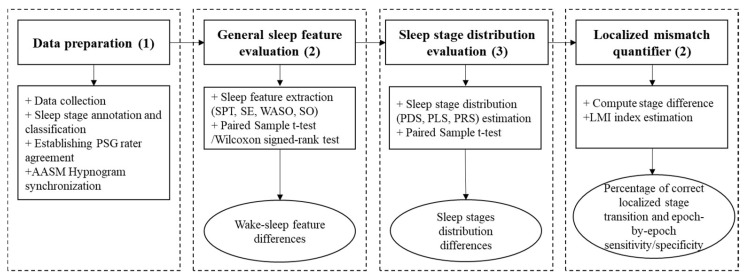
Overall description of the proposed evaluation framework.

The proposed evaluation framework consists of four steps: (1) data preparation, (2) wake-sleep feature evaluation, (3) sleep stage evaluation, and (4) localized mismatch quantification. The details of each step are described in Figure 1. In this figure, each vertical block describes the sequential steps, the corresponding tasks performed, and the resulting metrics for the evaluation of the PSG and sleep tracker device sleep staging mismatch. In the first step, the hypnograms from the wearable devices and PSG system were collected and synchronized. The sleep staging annotated by the sleep tracker device was synchronized with that annotated by PSG using the AASM sleep guideline. Therefrom, the general sleep features and sleep stage distribution were extracted and compared between PSG and wearable device hypnograms in the second and third step. The wake-sleep analysis, sleep staging distribution comparisons, and localized analysis were performed in sequence on the extracted sleep features and index to evaluate the sleep scoring performance of the wearable devices. Finally, the localized quantifier index was extracted, and the localized mismatch measured by the percentage of the correct transition was evaluated in step 4. The details of each step are described below.

### 2.1. Data Preparation

Data collected from the wearable devices and PSG system needs to be preprocessed for further comparison. In the initial step, the time synchronization of the signals from PSG and wearable devices was performed using the referenced clock time of the hosted computer. Next, the segmentation of the synchronized data into a 30-s epoch was implemented. This procedure is critical for the epoch-by-epoch (EBE) analysis and comparison. The sleep hypnogram–a graph that depicts sequential sleep stages in 30-s epoch over the sleeping time–from the PSG system was annotated by the sleep physician. The sleep hypnograms annotated by the wearable device’s algorithm were also segmented into the 30-s epoch. As multiple raters might be involved in the PSG scoring process, the Fleiss’ kappa statistic κ —the measurement to characterize the agreement at epoch level among the raters—is considered for testing the interrater reliability [27]. The Fleiss’ kappa is estimated as $\kappa =\left(\bar{P}-\bar{P_{e}}\right)/\left(1-\bar{P_{e}}\right)$, where $\bar{P}$ is the relative observed agreement among the raters and $\bar{P_{e}}$ is the probability of a chance agreement. If the raters completely agree upon the PSG hypnogram, κ=1, otherwise κ<1. The threshold of Fleiss’ kappa κ<0.8 is used to determine if the hypnogram should be concurrently revised and agreed upon among the raters before proceeding to the next step [23].

Sleep stage classifications among the wearable devices and the PSG are matched based on the American Academy of Sleep Medicine’s (AASM) guideline. Although the sleep stages, comprising of Wake, N1, N2, N3, and REM, were explicitly defined by AASM [24], not all commercial wearable devices interpret this sleep staging regulation consistently. The scoring of each PSG epoch to Wake, N1, N2, N3, and REM requires visual identification by professionally trained personnel. The expert interprets specific patterns (e.g., arousals, K-complexes, spindles) and tonic (e.g., percentage of slow-wave sleep within an epoch) features from the multiple EEG and physiological channels of PSG. The limitations of sensors and expertise interpretation have simplified the annotation of wearable sleep trackers. In particular, the wearable sleep trackers label sleep stages as Wake–Light–Deep or Wake–REM–Light–Deep. We annotated “Device type I” for the device group defining sleep stages into Wake–REM–Light–Deep and “Device type II” for the device group defining sleep stages into Wake–Light–Deep. In order to provide a fair comparison among wearable devices relative to PSG, light sleep is defined as N1 and N2, while deep sleep is N3 (in case of Device type I) or N3 and REM (in case of Device type II) as shown in Table 1.

### 2.2. General Features and Sleep Stage Distribution Evaluation

**Figure 2:**
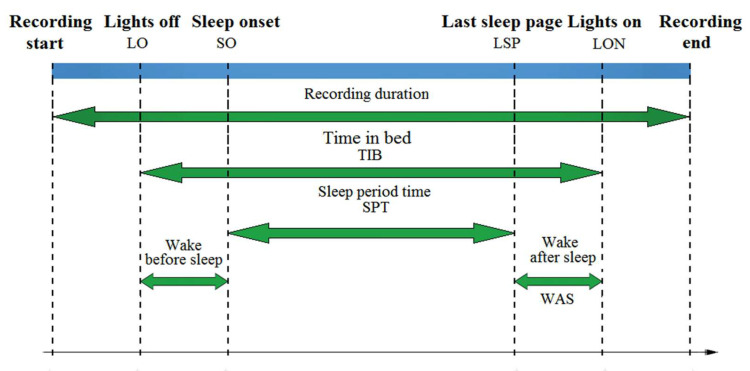
Timing diagram of a sleep data recording with the annotation of the significant sleep events and durations.

Two groups of features, namely the wake-sleep feature and sleep stage distribution, were extracted from wearable sleep trackers and PSG and were pairwise compared. The wake-sleep features comprise three subgroups—sleep quality, sleep disturbance, and wake-sleep transition. Sleep quality features characterize the influence of the mental, social, and physical impacts of sleep on human health [28]. Sleep disturbance and wake-sleep transition, on the other hand, quantify the temporal shifting between sleep and wake stages and are used mainly for the diagnosis of common sleep disorders such as insomnia, narcolepsy, and depression [29]. These features were estimated from the timing diagram (as shown in Figure 2) defining the onset and the offset of the significant events and the elapsed time from the start to the end of the data recording. The sleep stage distribution features characterize the duration and percentage of light, deep, and REM sleep over the total sleeping time. The alterations of these features are frequently used to screen for pathologically architectural changes in several sleep disorders such as insomnias, dyssomnias, parasomnias, sleep-related breathing, and circadian rhythm sleep disorders. The description and derivation of these features are summarized in Table 2. The comparative statistics of these features separate the aggregate differences of the wake-sleep patterns and sleep architectures annotated by wearable sleep trackers and the PSG system.

### 2.3. Localized Quantifier Index

A localized mismatch index (*LMI*) was proposed to characterize the epoch-to-epoch difference between the sleep stages annotated from PSG and those from wearable devices over the sleeping time. The *LMI* was estimated on the basis of comparing the annotated hypnograms derived from the PSG systems and the wearable devices. The hypnographic sequences of the wearable devices were denoted as $Y_{W}=\left\{Y_{W_{1}},Y_{W_{2}},Y_{W_{3}}\ldots Y_{W_{n}}\right\}$, and of the PSG system as $Y_{P}=\left\{Y_{P_{1}},Y_{P_{2}},Y_{P_{3}}\ldots Y_{P_{n}}\right\}$, where each sequence element was the designated values of the sleep stages at the i^*th*^ epoch and *n* was the number of epochs over the night. At first, a set of the epoch index at which the sleep stage transition in PSG and wearable device hypnograms were different was estimated. This set was calculated as $A=\left\{i|\Delta Y_{P_{i}}=Y_{P_{i+1}}-Y_{P_{i\;}\;}\neq 0\vee \Delta Y_{W_{i}}=Y_{W_{i+1}}-Y_{W_{i}}\neq 0\right\}$, where $Y_{P_{i\;}\;}$ and $Y_{W_{i}}$ were the hypnogram value of the PSG and the wearable device, respectively, and $\Delta Y_{P_{i}}$ and $\Delta Y_{W_{i}}$ were the PSG and wearable hypnogram difference at epoch *i*, respectively. The to characterize the correlations in the sleep stage transitions between the wearable device and PSG hypnograms at epoch *i^th^* was estimated as $LMI_{i}=\Delta \;Y_{W_{i}}\times \Delta Y_{P_{i}}$. Finally the percentage of correct PSG-wearable sleep stage transitions was computed based on the $LMI_{i}$ with the range of *i* from 1 to *n*.

Each component of the hypnographic sequence was assigned as $Y_{W_{i}}$ = 1 if the epoch was annotated as Wake, $Y_{W_{i}}$ = 2 as Light, and $Y_{W_{i}}$= 4 as Deep. Hence, all possible combinations of sleep stage transition in the hypnographic sequences were Wake to Light (W–L), Wake to Deep (W–D), Light to Deep (L–D), Light to Wake (L–W), Deep to Light (D–L) and Deep to Wake (D–W), and the resulted $\Delta Y_{P_{i}}$ and $\Delta Y_{W_{i}}$ of the sleep stage transition were 1, 3, 2, −1, −2, and −3, respectively. The possible localized mismatch index values of *LMI_i_* estimated from the $\Delta Y_{P_{i}}$ and $\Delta Y_{W_{i}}$ are presented in Table 3. It is noted that, as a consequence of the indices defined above, the table of $LMI_{i}$ values is symmetrical about the diagonal, as indicated by the yellow highlighted portions. The values in the diagonal line (i.e., 1, 4, and 9) indicate the full correlation between wearable devices and PSG while the off-diagonal values indicate the uncorrelated relationship. Accordingly, *LMI_i_* = 1, 4, and 9 denotes the correct detection of the wearable device for the Wake and Light, Light and Deep, and Wake and Deep transitions, respectively. If only the wake and sleep (ie., Light and Deep) transitions are considered, $LMI_{i}$ = 1 and 9 denotes the correct Wake transition, respectively, while $LMI_{i}$ = 4 denotes the correct Light/Deep transition. The other off-diagonal values including −9, −4, −6, −2, −3, −1, 2, 3, and 6 denote the other possible wrong detections of sleep stage transition. The algorithm was applied on each sleep tracker device if multiple devices were evaluated simultaneously. The implementation algorithm is presented in Table 4 and a detailed description of LMI for the evaluation of the type I sleep tracker with more sleep stages including Wake, Light, Deep, and REM is included in Table A1 in the Appendix A.

### 2.4. Statistical Test

The paired sample *t*-test, Wilcoxon signed-rank test, and Cohen’s d effect were used for the comparative tests. As the measurements from the PSG and wearable devices were taken on the same subjects at the same time, paired sample *t*-tests were performed to compare normally distributed features, while the Wilcoxon signed-rank test was performed for non-normally distributed features. The paired sample *t*-tests and Wilcoxon signed-rank test were utilized to test the null hypothesis that the sleep features from the PSG system are the same as those from the sleep tracker device. In addition, Cohen’s d effect size value annotated as η^2^ was used to measure the level of difference between the mean values of the sleep features detected from PSG and wearable devices. The larger the effect size, the more significant difference between the PSG and wearable devices. The significant level of *ρ* of 0.05 was used for all of the statistical tests. The confusion matrix of the sensitivity–the proportion of PSG-registered epochs also identified as the same sleep stages by the sleep tracker devices–, and specificity–the proportion of wake epochs correctly identified by the sleep tracker device–, were also considered.

## 3. Results

To highlight the salient features of the proposed framework, we evaluated four different sleep tracker devices’ performance with the PSG ground truth and compared the results with the other evaluation methods. The experimental setup and data collection for the evaluations are presented. Next, we explain the results of the aggregating sleep features and the localized mismatch quantifier of each sleep tracker, and the comparison among the four sleep trackers. The aggregating features of wake-sleep patterns and sleep architectures are demonstrated using the data obtained from the four sleep tracker devices. The chronologically epoch-to-epoch mismatch of the wearable sleep tracker devices and the PSG ground truth is described. Finally, the comparison of the proposed method and the existing evaluation methods is reported.

### 3.1. Experimental Setup and Data Collection

**Figure 3:**
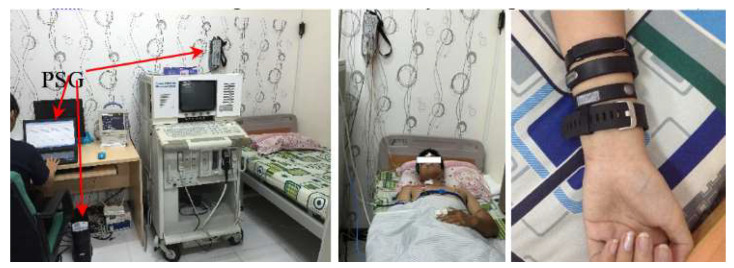
Setup of PSG system and wearable devices for each overnight data acquisition.

The proposed framework was demonstrated to validate the performance of four different wearable sleep tracker devices and the PSG system in a research sleep laboratory setting. The data collection was performed at the Sleep Lab of International University, Ho Chi Minh City, Vietnam National University (IU-VNU HCMC). The data was collected on 22 (14 males, 8 females) subjects in the age range of 18 to 36. The study IRB was approved by the research ethics committee of the IU-VNU HCMC. The polysomnographic data were collected using an Alice5 Philips™ PSG system. The setup of the polysomnography system and data acquisition is shown in Figure 3. In our setup, all of the measurements were collected on the subject’s left hand with a negligible difference in the rotating angle on the forearm’s volar (back) side. Furthermore, we set up the wearable device in the same oriented directions with the random placement order. With such a setup, we assumed that the sensor placements were comparable among the wearable devices. The PSG system consisted of 11 sensor types, including electroencephalogram (EEG), electrooculography (EOG), electrocardiography (ECG), leg electromyogram (EMG), chin EMG, thermal flow, snoring, chest, and abdomen respiration sensors, a positioning sensor, and a pulse oximeter. During the experiment, participants were also required to simultaneously wear the four types of wearable devices (described below) on their wrist, along with the PSG system.

Among the 72 overnight datasets collected, 42 were excluded because of the incompleteness of the collected signals (6 came from PSG problems, 32 from wearable devices, 4 from both). The exclusion criteria were: (1) missing overnight hypnogram collected by the wearable device because of the intermittent battery issue or unexpected bluetooth paring problem; (2) missing PSG signals for the sleep physician to determine the sleep stage caused by the movement of the subject during the experiment, which leads to the displacement or dislocation of PSG sensors, especially the EEG signals. To have the devices fairly compared, all devices were tested to ensure the proper functioning before the data collection and two physicians were available to monitor and readjust the dislocated sensors over the night. A set of 30 overnight datasets with approximately 8 sleeping hours per night were used for data analysis. The PSG data were interpreted and scored by a sleep physician using AASM scoring criteria [24]. Finally, the data were de-identified and stored anonymously for further analysis. To minimize the intrarater errors, the procedures to set up the device and to collect the data were standardized and performed by the trained technicians.

The commercial wearable sleep tracking devices with sleep monitoring functions were named as devices A, B, C, and D and compared anonymously. In detail, all four devices used accelerometer sensors to continuously record 3-axis translational wrist motion during the night. In addition to the accelerometers, Device B was equipped with an optical sensor to measure the heart rate, and Device C contained a bio-impedance sensor to measure heart rate, respiratory rate, body temperature, and galvanic skin response. All devices streamed data automatically onto an online server, from which the algorithms were used to classify sleep data into different sleep stages. Here, the sleep staging algorithms were developed by the wearable device companies and the device users were not permitted to intervene in the annotated staging. Devices A, B, and D are categorized as type II, whereas Device C is a type I device.

### 3.2. Wake-Sleep Analysis

The evaluation of wake-sleep features from four wearable sleep trackers benchmarking with the PSG system was investigated. Here, as the measurements from PSG and wearable devices were concurrently taken on the same subject, the paired sample *t*-tests were performed to compare the normally distributed features of SPT and SO, and Wilcoxon signed-rank tests were performed for non-normally distributed features of WASO and SE. The null hypothesis is that the mean/median of sleep features from the wearable devices and those from the PSG are equal. The differences were significant based on the value of *ρ* and the power of the test *η*^2^. On the basis of these criteria, Table 5 demonstrates the comparative results of each device with the PSG system over four different wake-sleep features column-wise, and among four different devices row-wise. As noted, Device C was the most comparable to PSG. Specifically, there was no significant difference between the SE and WASO features of Device C and those of the PSG. Even though significant differences were found for the SPT and SO features of Device C relative to the PSG, it is important to note that those tests had the lowest *η*^2^ values relative to the other comparative pairs. Devices D and A were ranked after Device C. Of all the devices, Device B showed the highest discrepancies for both SE and WASO features benchmarked to the PSG system, in terms of the number and strength of the significant differences obtained. The SPT and SO features of Device B also had significant statistical differences relative to PSG, but evidently not as strong.

### 3.3. Sleep Stage Distribution Evaluation

The evaluation of the sleep stage distribution demonstrated the agreement of sleep architectures from the four wearable sleep trackers and the PSG ground truth were investigated. Table 6 summarizes the comparative results of sleep distribution features from the four different wearable devices and the PSG system. Devices A, B, and D were categorized as type II sleep tracker devices with the sleep stages of Wake–Light–Deep, while Device C was categorized as type I with Wake–Light–REM–Deep stages. Hence, in addition to the PLS and PDS features, the sleep distribution features of type I devices also include the PRS feature. These differences were significant based on the value of *ρ* and the power of the test *η*^2^. From the testing results, the PRS feature in Device C, and the PLS and PDS features in Device A yielded an insignificant difference between the PSG and wearable devices. Although Device C detected the REM stage well, it was not able to provide good Light and Deep sleep detection, as shown in the testing results of the PLS and PDS features.

### 3.4. Localized Mismatch Analysis

**Figure 4:**
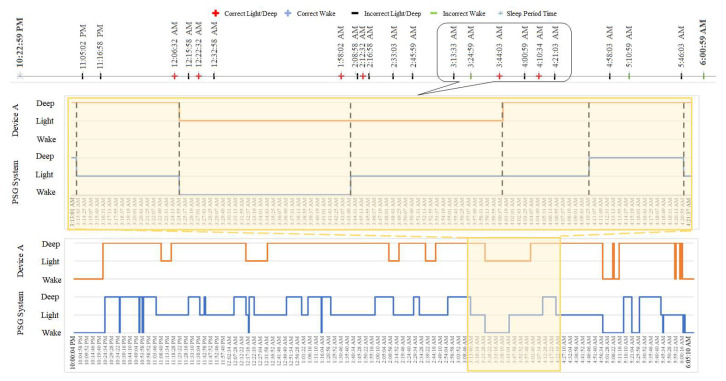
Representative PSG and Device A’s hynograms of a subject with the mismatch from 3:13 to 4:21 a.m.

The localized mismatch of the sleep staging annotated by the weareable sleep tracker devices with PSG benchmark was reported. Figure 4 demonstrates the hypnograms from the PSG and wearable devices and the corresponding estimated LMI from a demonstrative dataset. The lower graph describes the hypnograms of PSG system and wearable device from 10:22 p.m. to 6:00 a.m., a zoomed-in portion of the hypnograms from 3:13 to 4:21 a.m. is highlighted in the middle graph. The LMI values were grouped into four groups. The epochs with different possible transitions include correct wake (LMI = 1, 9), correct sleep (LMI= 4), incorrect wake (LMI= 2, 6,−1,−2,−3,−6,−9), and incorrect sleep (LMI=3, −4), and are presented in the upper subgraph. The LMI vectore was estimated using the presented algorithm. Each element i of the LMI vector quantifies a sleep stage transition and the value $LMI_{i}$ describes the sleep stage difference of PSG and the sleep tracker device’s hypnograms. It should be noted that the LMI vector and the variation of the $LMI_{i}$ values quantify repspectively the chronological mismatch and the distribution of them over the data collection of the PSG and sleep tracker devices.

**Figure 5:**
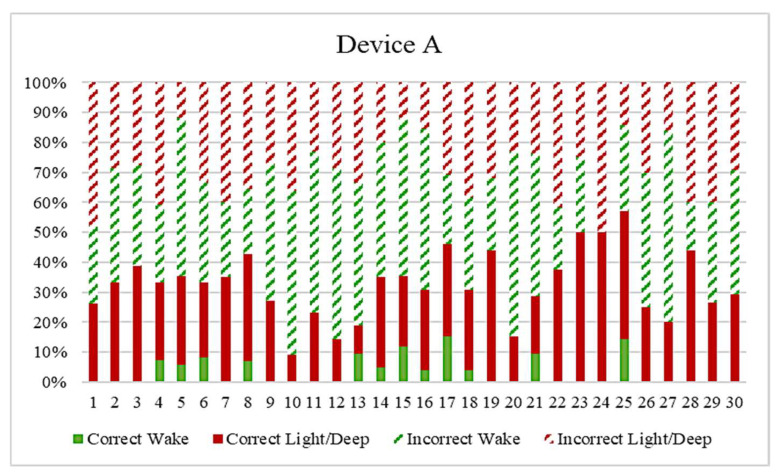
Reported results for the correct and incorrect sleep and wake stage transition in 30 datasets collected by Device A.

The percentage of correct and incorrect transition rates within the collected datasets and compared wearable devices were reported. Figure 5 presents the percentage of the correct and incorrect stage transitions of 30 datasets collected by Device A. It is noted that the overall incorrect rate (striped portions) outweigh the correct rate (solid portion). This confirmed the findings that the wearable devices were poor in capturing sleep stage transition, especially in the wake stage transition shown as the solid green portion in the figure. A summary of the percentage of correct and incorrect stage transitions for the four wearable trackers is reported in Table 7. This table can be used to compare the sleep stage scoring performance among the different wearable devices benchmarking to the PSG system.

A detailed sleep stage annotated by the wearable devices benchmarked to the PSG in all sleep stages is reported in Table 8. The sensitivity and specificity of each sleep stage were used to quantify the accuracy of the epoch-to-epoch correlation of the sleep tracker device and the PSG’s hypnograms. It should be noted that the wearable devices were capable of detecting the awake stage with very high specificity; however, they also overestimated the awake stage, as shown by low sensitivity. The imbalance between sensitivity and specificity is also prevalent in the Light, Deep, and REM sleep stages. Among the devices, Device B was distinctive because of the unbalanced sensitivity and specificity of detecting the Deep sleep stage (over 98% for sensitivity and under 10% for specificity), which is attributed to the tendency to annotate all sleep times as the Deep sleep stage. On the contrary, the other devices were more likely to classify sleep as the Light sleep stage. The overall accuracy of all devices was low, which quantitatively supports the premise that there is likely discrepancy between wearable devices and the PSG in scoring sleep stages.

### 3.5. Comparison with Other Methods

In this section, we compare the performance of the proposed method with three other methods, including Pearson correlation [19,22], two-way repeated ANOVA [19,22], and Tryon’s approach [18,22]. To make it comparable, the other methods were also used to assess the four wearable sleep trackers with the PSG on the basis of the same dataset as described in Section 3.1. The ranking of the device accuracy benchmarking to the PSG system is reported in Table 9. Accordingly, the results obtained from evaluating the general sleep features among different evaluation methods were quite similar. The three methods agreed that Device C gave the closest outcome to the PSG system, and Devices A and B yielded the least accuracy. For the SPT feature, however, while our method showed that Device D was the least comparable to PSG, the result from the Pearson correlation test ranked Device D as the second most accurate device. For the evaluation of the sleep distribution features, we compared our method with the Tryon’s approach. Table 10 shows two epoch-by-epoch comparisons, one using a confusion matrix for the multiple classes used in this study, and the other as a confusion matrix for the two classes of wake and sleep presented in Tryon’s approach [18,22]. The proposed method produced the same results in the wake-sleep sensitivity and specificity. However, instead of considering the REM, Light, and Deep stages as sleep, our proposed method was more advanced in providing the evaluating and ranking according to the discrepancy of each device in a detemined evaluated sleep stage.

## 4. Discussion and Conclusions

Our results from the proposed framework confirmed the findings from previous studies that, compared to the PSG ground truth, wearable sleep tracker devices effectively detect sleep onset and sleep period time, but are deficient in estimating N1, N2, N3, and REM stages [31]. The evaluation results highlighted the accuracy of actigraphy devices in detecting the wake-sleep stage. Although they perform well in detecting wake-sleep patterns, actigraphy wearable devices are not unified in defining the sleep stage as shown in results in the sleep staging distribution comparision. In particular, Devices A, C, and D seemed to define N1 and N2 as Light sleep, and N3 and REM as Deep sleep; meanwhile, Device B gave the best result when considering N1 as Light sleep and N2, N3, and REM as Deep sleep, which is still not quite suitable for the diagnosis of a sleep disorder in a clinical setting.

Moreover, on the basis of our evaluation, we found that the wearable devices supplemented by cardiorespiratory sensors rather than actigraphy data yield better sleep staging results. In particular, the results showed that among the four wearable devices, Device C with its bio-impedance sensor was the best one for accurately detecting both sleep-wake patterns and REM sleep. It is inferred that an appropriate way to define sleep stages is by especially considering the integrated bio-signal data (i.e., heart rate, respiration rate, skin impedance, body temperature) in scoring sleep, which can enhance sleep scoring accuracy in wearable sleep tracker devices [32,33]. The localization quantifiers can be used to evaluate the effectiveness of the bio-signal sensors (i.e., heart rate, respiration, skin impedance, body temperature sensors) integrated within wearable devices in scoring sleep stages. Hence, the use of our proposed method is recommended for wearable devices with the inclusion of new bio-sensors that enable the detection of sleep stages defined by the AASM guildelines. One of the limitations of the proposed method is the constrain of the concurrent sleep staging and agreement among sleep raters on the PSG hypnogram if the Fleiss’ Kappa’s exceeds the threshold. Future work should consider multiple sleep staging from independent sleep raters and the correspoinding sensitivity of the local sleep mismatch index.

This paper proposed a method to assess and evaluate the sleep stage scoring in wearable sleep trackers with PSG ground truth. The framework’s three major steps consist of an AASM-based sleep stage classification, aggregating sleep feature evaluation, and localized mismatch evaluation. Overall, our study provides a comprehensive assessment tool for the evaluation and validation of sleep tracking devices benchmarking with the PSG system. Significantly, wake-sleep analysis results using a paired sample *t*-test are in line with previous results from the ANOVA and the Pearson correlation. Besides, our proposed quantifier is more advanced than the previous method, able to characterize the local mismatch between the hypnogram of PSG and sleep tracker devices. Besides the same results for wake-sleep sensitivity and specificity, it can evaluate the chrological difference over other sleep stages, such as REM, Light, and Deep sleep. Furthermore, the proposed method outweighed the other methods in tracking the sleep stage transition of REM, Light, and Deep sleep. By simultenously comparing results among different devices, the proposed method can be used to evaluate the effectiveness of the add-on bio-signal sensors (i.e., heart rate, respiration, skin impedance, body temperature sensors) integrated within the wearable devices in scoring sleep stages. The proposed method provides a more detailed and systematic procedure to assess and validate the emerging point-of-care polysomnographic alternatives.

## Figures and Tables

**Table 1:** Methods of categorizing sleep staging scored by wearable devices and the corresponding sleep stages according to AASM guidelines.

		Sleep Stages				
	PSG	Wake	N1	N2	N3	REM
Wearable Devices						
Type I		Wake	Light		Deep	REM
Type II		Wake	Light		Deep	

**Table 2:** Derivation of wake-sleep features and sleep stage distribution from the timing features defined in Figure 2**.**

Category		Features	Description	Formula	Unit
		Total sleep time (TST)	Time spent on sleeping during sleep period time (SPT)	TST = TLST + TDST + TRST	min
Wake-sleep features	Sleep quality	Sleep period time (SPT)	Elapsed time from sleep onset (SO) to last epoch of sleep (LSP)	SPT = LSP-SO	min
		Sleep efficiency (SE)	Ratio of total sleep time (TST) to sleep period time (SPT)	SE = TST/SPT	%
	Sleep disturbance	Wakefulness after sleep onset (WASO)	Awake time (AWT) during sleep onset (SO) to lights on (LO) [30]	WASO = SPT-TST+WAS	min
	Wake-sleep transition	Sleep onset (SO)	The point of time when the subject undergoes a transition from wakefulness into sleep.	1st N1/N2/N3/REM	hh:mm:ss
Sleep stage distribution		Percentage of light sleep (PLS)	Ratio of total light sleep time (TLST) over total sleep time (TST)	PLS = TLST/TST	%
		Percentage of deep sleep (PDS)	Ratio of total deep sleep time (TDST) over total sleep time (TST)	PDS = TDST/TST	%
		Percentage of REM sleep (PRS)	Ratio of total REM sleep time (TRST) over total sleep time (TST)	PRS = TRST/TST	%

**Table 3:** The localized mismatch index values between wearable Device type II and PSG with “W” annotated for Wake, “L” for Light, “D” for Deep, and the operation ‘-‘ for the transition between sleep stage (i.e., W–L is Wake to Light transition).

LMI Value	Device Type II		W–L	W–D	L–D	L–W	D–L	D–W
PSG		∆Y_W_	1	3	2	−1	−2	−3
	∆Y_P_							
W-L	1		1	3	2	−1	−2	−3
W-D	3		3	9	6	−3	−6	−9
L-D	2		2	6	4	−2	−4	−6
L-W	−1		−1	−3	−2	1	2	3
D-L	−2		−2	−6	−4	2	4	6
D-W	−3		−3	−9	−6	3	6	9

**Table 4:** Implementation of the proposed evaluation.

-Synchronize and align the data using clock time of the host computer -Specify the AASM sleep scoring category of the wearable device -Establish agreement among sleep raters on the PSG hypgram -General features and sleep stage distribution evaluation +Extract general sleep features +Extract sleep state distribution features -Localized quantifier estimation +Construct the hypnographic states of wearable device $Y_{W}=\left\{Y_{W_{1}},Y_{W_{2}},Y_{W_{3}}\ldots Y_{W_{n}}\right\}$ +Construct the hypnographic states of PSG $Y_{P}=\left\{Y_{P_{1}},Y_{P_{2}},Y_{P_{3}}\ldots Y_{P_{n}}\right\}$ +Compute sleep state difference $\Delta Y_{P}$ and $\Delta Y_{W}$ +Detect epochs with sleep stage transition as A +Estimate sleep stage correlation indexes $LMI_{i}$ +Determine the correct and incorrect sleep transition epoch based on the $LMI_{i}$ -Overall evaluation +Estimate *ρ* and *η*^2^ for wake-sleep features and sleep stage distribution comparison +Estimate the percentage of the correct sleep stage transition +Estimate sensitivity, specificity, and accuracy from percentage of correct transition of each sleep stage.

**Table 5:** Summary of *ρ* and *η*^2^ values for paired sample *t*-tests performed on wake-sleep features.

Features	PSG and Device A	PSG and Device B	PSG and Device C	PSG and Device D
SPT	(*)*ρ* = 0.005 *η*^2^ = 0.572	(*)*ρ* = 0.001 *η*^2^ = 0.717	(*)*ρ* = 0.027 *η*^2^ = 0.297	(*)*ρ* < 0.001 *η*^2^ = 0.782
SE	(*)*ρ* = <0.001 *η*^2^ = 4.613	(*)*ρ* <0.001 *η*^2^ = 5.566	(*)*ρ* = 0.039 *η*^2^ = 2.301	(*)*ρ* = 0.001 *η*^2^ = 3.365
WASO	(*)*ρ* <0.001 *η*^2^ = −19.508	(*)*ρ* <0.001 *η*^2^ = −24.017	(*)*ρ* = 0.032 *η*^2^ = −9.492	(*)*ρ* = 0.002 *η*^2^ = −14.500
SO	(*)*ρ* <0.001 *η*^2^ = 0.083	(*)*ρ* <0.001 *η*^2^ = 0.083	(*)*ρ* = 0.042 *η*^2^ = 0.034	(*)*ρ* = 0.001 *η*^2^ = 0.079

“(*)” indicates significant difference between the device and PSG system, for a significance level *ρ* = 0.05. The power of the test *η*^2^ indicates the separation between sleep features from the wearable devices and those from the PSG; the larger the *η*^2^, the more different the device and the PSG.

**Table 6:** Statistical comparison of Light, Deep, and REM stages among four sleep tracker devices.

	Device A (Accelerometer Sensor) and PSG	Device B (Accelerometer Sensor, Optical Sensor) and PSG	Device C (Accelerometer sensor, Bio-Impedance Sensor) and PSG	Device D (Accelerometer Sensor) and PSG
PLS	(**) *ρ* = 0.254 *η*^2^ = 0.250	(*) *ρ* < 0.001 *η*^2^ = 6.638	(*) *ρ* = 0.001 *η*^2^ = 0.898	(*) *ρ* = 0.002*η*^2^ = 0.853
PDS			(*) *ρ* < 0.001 *η*^2^ = 1.373	
PRS			(**) *ρ* = 0.820; *η*^2^ = 0.057	

“(**)” means no significant difference between the wearable device and PSG system. “(*)” indicates significant difference between the device and PSG system, for significance level *ρ* = 0.05. The power of the test *η*^2^ indicates the separation between sleep features from the wearable devices and those from PSG; the larger the *η*^2^, the more different the device and the PSG.

**Table 7:** Correct and incorrect sleep stage transition rates among the four wearable devices.

	Correct Transition Rate	Incorrect Transition Rate
Device A	31.83%	68.17%
Device B	24.35%	75.65%
Device C	24.66%	75.34%
Device D	28.86%	71.14%

**Table 8:** Confusion matrix of epoch-by-epoch comparison among the four wearable devices and the PSG’s hypnograms for the four sleep stages of Wake, Light, Deep, and REM.

Wearable Devices	Wake		Light		Deep		REM		Acc.
	Sen.	Spec.	Sen.	Spec.	Sen.	Spec.	Sen.	Spec	
Device A	9.24	99.17	55.94	43.75	50.91	64.36			65.10
Device B	3.39	99.92	2.87	92.97	98.70	8.71			61.75
Device D	27.88	98.57	41.40	62.10	65.76	51.63			66.38
Device C	40.17	95.54	73.30	47.81	28.07	92.93	25.01	84.51	40.17

**Table 9:** Comparison among different evaluation methods and the proposed method on evaluating the general sleep features with the ranking according to the accuracy with PSG.

General Sleep Feature Evaluation	Proposed Method (*ρ* Statistic)	Pearson Correlation (r Statistic) [19,22]	Pairwise Comparison (Two-Way ANOVA) [19,22]
SPT	Device C, A, B and D	Device C, D, A and B	Device C, A, D and B
SE	Device C, D, A and B	Device C, D, A and B	Device C, D, A and B
WASO	Device C, D, A and B	Device C, D, A and B	Device C, D, A and B
SO	Device C, D, A and B	Device C, D, A and B	Device C, D, B and A

**Table 10:** Comparison between the proposed method and Tryon’s approach on evaluating sleep distribution feature with the ranking according to the accuracy with PSG.

Sleep Distribution Evaluation	Proposed Methods	Tryon’s Approach [18,22]
Light	Device A, D, C and B	Device C, D, A and B
Deep	Device A, D, C and B	
REM	Not significantly different	

## Appendix A

**Table A1:** Detailed *LMI* table for the evaluation of the type I sleep tracker. The localized mismatch index values between wearable device type I and PSG with “W” annotated for Wake, “R” for REM, “L” for Light, “D” for Deep and the operation ‘-‘for the transition between sleep stages (i.e., W-L, Wake to Light transition).

*LMI* Value	Device Type I		W–R	W–L	W–D	R–L	R–D	L–D	R–W	L–W	D–W	L–R	D–R	D–L
PSG		∆Y_W_	1	3	8	2	7	5	−1	−3	−8	−2	−7	−5
	∆Y_P_													
W-R	1		1	3	8	2	7	5	−1	−3	−8	−2	−7	−5
W-L	3		3	9	24	6	21	15	−3	−9	−24	−6	−21	−15
W-D	8		8	24	64	16	56	40	−8	−24	−64	−16	−56	−40
R-L	2		2	6	16	4	14	10	−2	−6	−16	−4	−14	−10
R-D	7		7	21	56	14	49	35	−7	−21	−56	−14	−49	−35
L-D	5		5	15	40	10	35	25	−5	−15	−40	−10	−35	−25
R-W	−1		−1	−3	−8	−2	−7	−5	1	3	8	2	7	5
L-W	−3		−3	−9	−24	−6	−21	−15	3	9	24	6	21	15
D-W	−8		−8	−24	−64	−16	−56	−40	8	24	64	16	56	40
L-R	−2		−2	−6	−16	−4	−14	−10	2	6	16	4	14	10
D-R	−7		−7	−21	−56	−14	−49	−35	7	21	56	14	49	35
D-L	−5		−5	−15	−40	−10	−35	−25	5	15	40	10	35	25
